# Critical scattering and incommensurate phase transition in antiferroelectric PbZrO_3_ under pressure

**DOI:** 10.1038/srep41512

**Published:** 2017-01-30

**Authors:** R. G. Burkovsky, I. Bronwald, D. Andronikova, B. Wehinger, M. Krisch, J. Jacobs, D. Gambetti, K. Roleder, A. Majchrowski, A. V. Filimonov, A. I. Rudskoy, S. B. Vakhrushev, A. K. Tagantsev

**Affiliations:** 1 Peter the Great Saint-Petersburg Polytechnic University, 29 Politekhnicheskaya, 195251, St.-Petersburg, Russia; 2 Ioffe Institute, 26 Politekhnicheskaya, 194021, St.-Petersburg, Russia; 3 Department of Quantum Matter Physics, University of Geneva, 24, Quai Ernest Ansermet, 1211 Genéve 4, Switzerland; 4 Laboratory for Neutron Scattering and Imaging, Paul Scherrer Institute, 5232 Villigen PSI, Switzerland; 5 European Synchrotron Radiation Facility, BP 220, F-38043 Grenoble Cedex, France; 6 Institute of Physics, University of Silesia, ul. Uniwersytecka 4, 40-007 Katowice, Poland; 7 Institute of Applied Physics, Military University of Technology, ul. Kaliskiego 2, 00-908 Warsaw, Poland; 8 Ceramics Laboratory, Swiss Federal Institute of Technology (EPFL), CH-1015 Lausanne, Switzerland

## Abstract

Antiferroelectric lead zirconate is the key ingredient in modern ferroelectric and piezoelectric functional solid solutions. By itself it offers opportunities in new-type non-volatile memory and energy storage applications. A highly useful and scientifically puzzling feature of this material is the competition between the ferro- and antiferroelectric phases due to their energetic proximity, which leads to a challenge in understanding of the critical phenomena driving the formation of the antiferroelectric structure. We show that application of hydrostatic pressure drastically changes the character of critical lattice dynamics and enables the soft-mode-driven incommensurate phase transition sequence in lead zirconate. In addition to the long known cubic and antiferroelectric phases we identify the new non-modulated phase serving as a bridge between the cubic and the incommensurate phases. The pressure effect on ferroelectric and incommensurate critical dynamics shows that lead zirconate is not a single-instability-driven system.

Antiferroelectric (AFE) crystals are of high technological importance. They are principal ingredients in lead zirconate-titanate solid solutions that are the most widely used materials for electromechanical transducers and actuators, as well as for ferroelectric random access memory and electrooptical devices^1^,^2^,^3^. Antiferroelectrics themselves are prospective in energy storage, large-displacement actuators and high-density non-volatile memory based on the effect of ferroelectricity in the AFE domain walls^4^,^5^. The competition between ferro- and antiferroelectric structures in antiferroelectrics is at the heart of their practical importance. In contrast to ferroelectric phases, for which the formation mechanisms *via* the soft mode condensation are well assessed and understood^6^, the formation of AFE phases is presently a matter of hot debate^7^,^8^,^9^,^10^,^11^. In the absence of direct experimental data, a common assumption was that the anti-polar shifts of Pb^2+^ ions in the most studied antiferroelectric PbZrO_3_ occur due to condensation of specific soft AFE phonon mode at the relevant wavevector 
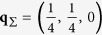
12,13, in analogy with the formation of the ferroelectric (FE) phase in ferroelectrics *via* the softening of the Γ-point (**q**_Γ_ = (0, 0, 0)) optic phonon^14^,^15^. However, the first experimental study of the lattice dynamics in PbZrO_3_ revealed a transverse acoustic (TA) phonon branch that is strongly flattened at finite wavevectors and sensitive to temperature changes, but without minima at **q**_∑_ or any other wavevector along the ∑(*ξ, ξ*, 0) direction^7^. The absence of minima makes a range of wavevectors along the ∑ direction appearing “equally favourable” in the sense of a possible formation of a modulated structure that may be commensurate or incommensurate (IC). The apparent possibility of forming an IC structure indicates that the antiferroelectricity can be understood as the lock-in phase in an IC phase transition sequence, in which the IC structure itself is omitted due to the strong anharmonic interactions favouring premature phase locking^7^. Antiferroelectricity in PbZrO_3_ can thus be interpreted as a “missed” incommensurate phase. Indeed, the IC phases were not identified in PbZrO_3_ single crystals, in contrast to their common appearance in chemically doped compounds^16^. In exception to this trend stands the observation of IC satellites in PbZrO_3_ ceramics^17^, which was not confirmed by x-ray studies in single crystals^7^. This implies that the IC phase could be due to defects or microstrain specific to the ceramics.

The newly identified character of the critical dynamics suggests that the IC phases might indeed be found in PbZrO_3_ provided that the energetics of the crystal was renormalized in a way that provides a maximum of generalized susceptibility at a finite wavevector *q* as, for example, in classical IC crystals like K_2_SeO_4_^18^. Two different models were recently proposed to explain the observed flat and temperature-dependent generalized susceptibility landscape in PbZrO_3_ in the ∑ direction. In ref. 7 it was suggested that the observed dynamics is due to the flexoelectric interaction that couples the ferroelectric transverse optic (TO) branch to the TA branch, making the latter soft and temperature-dependent, in analogy with the behavior in KTaO_3_^19^,^20^. An alternative model was proposed in ref. 8, where the lattice dynamics in the ∑ direction is understood in terms of an intrinsically flat soft phonon branch. Being conceptually different, both models link the complex critical lattice dynamics to the single temperature-dependent, critical parameter – the stiffness of the zone-center FE soft mode *α*(*T*). A problem arises with this approach when the second thermodynamical variable, pressure, *p* is taken into account. From a set of high-pressure-high-temperature studies of the macroscopic properties of perovskites^12^,^21^,^22^, including PbZrO_3_^12^,^13^, it is known that the zero wavevector ferroelectric (FE) soft mode becomes “harder” on pressure increase, while the finite-wavevector lattice modes should become “softer”, as it appears from the pressure dependence of the AFE transition temperature^12^. From this point of view it turns out that a single critical parameter *α*(*p, T*) that strongly depends on the external conditions may be not enough for assessing phase transitions in PbZrO_3_ in the pressure-temperature space.

Despite a strong attention to PbZrO_3_ and the fact that some of the above or related questions were raised many years ago^12^, no direct experimental clarification on the microscopic level has been made. In order to obtain the relevant answers a study of critical dynamics at finite wavevectors by means of x-ray or neutron scattering under simultaneous application of high temperature and hydrostatic pressure is required. The corresponding experimental techniques are sufficiently challenging and no reports of their application in the context of perovskite ferro- and antiferroelectrics are presently available. We report on the results of the first experiment of this type on PbZrO_3_ using diffuse and inelastic x-ray scattering, performed at the European Synchrotron Radiation Facility. These experiments enabled us appending the pressure-temperature phase diagram of this long-known material with the two new phases linked to each other by an incommensurate phase transition. Our findings provide the ground for reassessment of the theoretical models explaining the mechanisms of antiferroelectric phase transitions.

## Results

**Figure 1:**
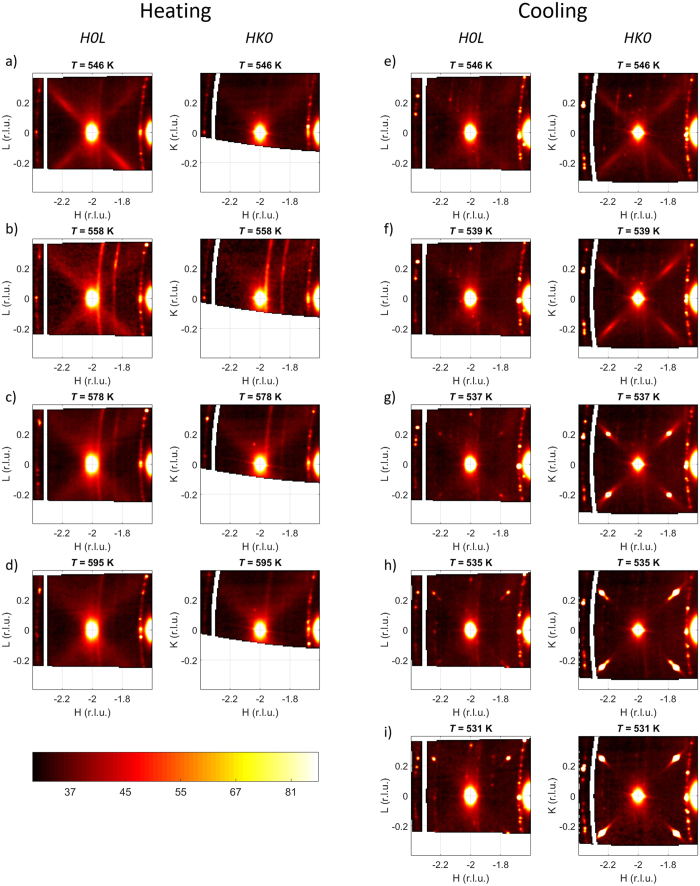
Distribution of x-ray scattering intensity in *H*0*L* and *HK*0 reciprocal space planes at *p* = 13 kbar. Panels (a–d) correspond to the heating cycle, panels (e–i) – to the subsequent cooling cycle. Panels (a,b,e,f) correspond to the new non-modulated phase, panels (c,d) – to the cubic phase, panels (g–i) – to the incommensurate and antiferroelectric phases. The strong diffuse scattering at the zone center is due to acoustic phonons. The position and magnitude of the large parasitic spot at **Q** ≈ (−1.6, 0, 0) is compatible with rhenium oxide (see Methods), that may grow on the sample surface at high temperatures. The distributions were obtained by a custom 3D reconstruction program.

We have studied PbZrO_3_ single crystals by complementary diffuse and inelastic x-ray scattering experiments. The measurements were performed at ID28 of the ESRF in a wide temperature range at pressure *p* = 13 kbar. The pressure value was selected as a representative pressure above the crossover separating the regions with and without pronounced peak in the temperature dependence of dielectric permittivity 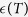, as known from refs 12, 13. Despite the notable change in 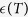 at *p* = 13 kbar compared to ambient pressure, no additional phase transitions were reported on the basis of the available dielectric data^12^. Contrary to that, our x-ray scattering results show a new phase in the temperature range from *T* = 539 K up to 559–578 K. Figure 1 shows the x-ray scattering distributions obtained in the heating and cooling cycles in different phases, including the new phase, which is represented by panels (a,b,e,f). The distribution of diffuse scattering (DS) in these panels indicates that the symmetry of the crystal is lower than cubic, for which evidences the difference in the DS distributions in the *H*0*L* and *HK*0 (in pseudocubic notation) reciprocal space planes that shall be equivalent in a cubic crystal. In the heating cycle, at *T* = 546 K (panels (a,b)), the strong DS signal is present in the *H*0*L* plane and not present in the *HK*0 plane while on cooling at *T* = 539 K (panels (e,f)) the strong DS signal is in *HK*0 plane and not in *H*0*L* plane.

The symmetry characterizing the actual DS distributions indicates that the symmetry of the new phase is at least as low as tetragonal. The apparently present difference in the DS intensity at **q** ≈ (−0.2, 0, 0.2) and **q** ≈ (0.2, 0, 0.2) in panels (a) and (b) allows suggesting that the symmetry of this phase may be as low as orthorhombic. This assumption is supported by the splitting of the pseudocubic reflections due to the presence of different low-symmetry domains (see Supplementary Materials). The magnitude of splitting indicates that the new non-cubic phase is characterized by about 1.5 times smaller pseudocubic unit cell distortions as compared to the antiferroelectric phase. We were lucky to obtain different domain states of this crystal structure on heating and cooling runs which rules out the possibility of explaining the observed DS asymmetry as an experimental artefact. The cubic symmetry is restored on heating between *T* = 559 K, where the asymmetry in the DS distribution is present, and *T* = 578 K, where the distribution is characterized by cubic symmetry.

The newly found non-cubic high-temperature phase in PbZrO_3_ is peculiar. On one hand there are no superstructures along the (hereafter – pseudocubic) ∑ directions that implies the absence of AFE modulations or other modulations with arbitrary wavevector. On the other hand the distributions display maxima of diffuse scattering (DS) intensity at finite wavevectors. This is very unusual for a simple perovskite crystal. The simple perovskite ferro- and antiferroelectrics display critical DS maxima at the zone center, as in BaTiO_3_^23^, KNbO_3_^24^, and PbTiO_3_^25^. Lead zirconate at ambient pressure also displays a maximum of critical DS at the zone center in the cubic phase, at temperatures just above the AFE transition. The shift of diffuse maximum from the zone center to finite wavevectors is unexpected and surprising.

**Figure 2:**
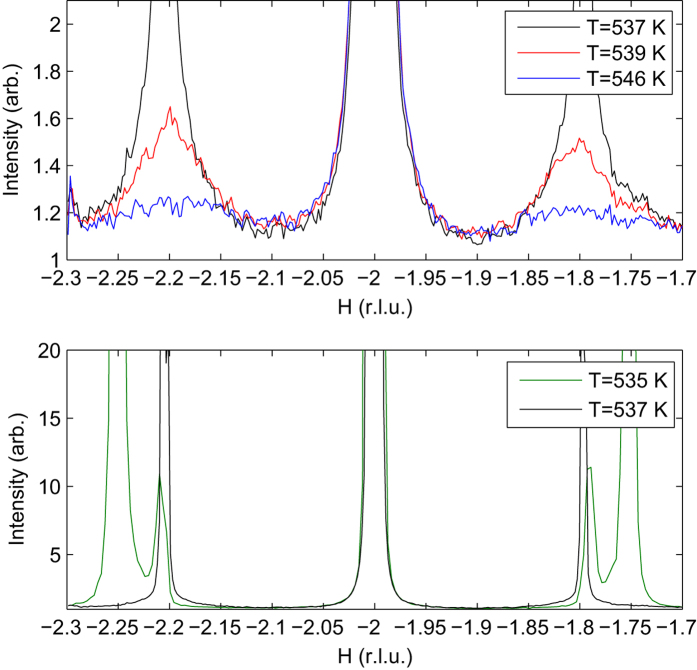
Profiles of energy-integrated scattering signal at *p* = 13 kbar along the Γ-M (−2 + *ξ, ξ*, 0) direction, in which the superstructures develop. The upper panel shows the increase of critical diffuse scattering at an incommensurate position on cooling and the formation of the long-range ordered incommensurate phase. The lower panel shows the evolution of the IC modulation wavevector on temperature decrease and the formation of the antiferroelectric modulation in the course of the first-order phase transition where IC and AFE phases coexist.

The new non-cubic phase is a bridge to the IC phase which was “missed” at ambient pressure but is found in this study under pressure. The precursor to the IC phase formation is the strong increase of the diffuse scattering intensity at finite wavevectors in the non-cubic non-modulated phase. This increase is seen in the upper panel of Fig. 2 which depicts the temperature evolution of the DS intensity on cooling along that ∑ direction which contains a maximum. The curves at *T* = 546 K and *T* = 539 K correspond to the case where the finite wavevector order parameter exists only in the form of fluctuations, without any long range order, while the curve at *T* = 537 K shows the long-range IC order already established. To demonstrate that, this curve is replicated in different scale in the lower panel of Fig. 2 where the sharp satellite IC Bragg reflections are clearly evident. The region of stability of this long-range IC modulation is quite narrow – about 4 K. We have taken only two diffraction datasets in this range. The second dataset, corresponding to *T* = 535 K, shows the IC phase already in the process of its replacement by the commensurate AFE modulation with wavevector 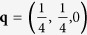 in the course of the first-order phase transition where the two phases coexist. The IC modulation wavevector changes from 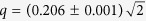 at *T* = 537 K to 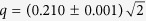 at *T* = 535 K. The IC superstructures, as well as the AFE superstructures, develop solely at the positions where the DS maxima were observed in the higher-temperature phase and not in the places where there were no maxima (excluding the very minor peaks in the *H*0*L* plane as compared to the ones in the *HK*0 plane in Fig. 1). This clearly confirms the link between the observed IC critical diffuse scattering and the formation of IC and AFE phases.

The sequence of phase transitions in PbZrO_3_ on cooling from non-modulated phase to the AFE phase allows recognizing the classical soft-mode-driven IC phase transition scenario consisting from high-temperature, IC and lock-in phases^26^,^27^. In ref. 7 it was suggested that the AFE phase corresponds to the lock-in phase. Our present data show that the formation of the IC phase is also consistent with the soft-mode-driven IC phase transition scenario. The increase of DS intensity on approaching the phase transition temperature indicates the critical slowing down of the relevant order parameter fluctuations^27^. These fluctuations may and are expected to be associated with the soft phonon dynamics. However, from the DS alone it is not possible to characterize the energy scale of this dynamics. In order to achieve that we applied *in-situ* inelastic x-ray scattering (IXS) characterization using the spectrometer of ID28. This type of measurements is very time-consuming and we were able to take only one IXS dataset in the non-cubic and non-modulated phase, sufficiently close to the IC phase transition temperature, at *T* = 546 K.

**Figure 3:**
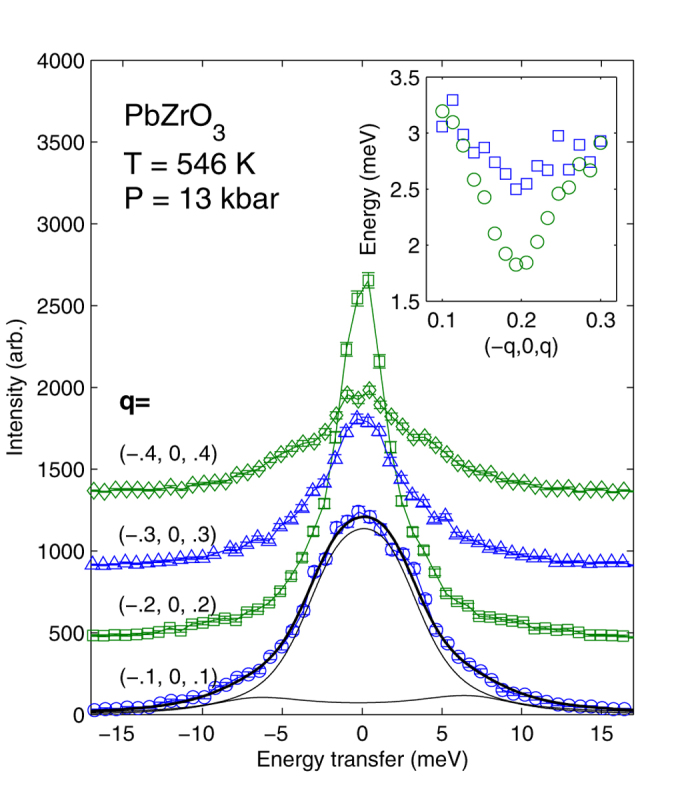
Inelastic x-ray scattering spectra for different wavevectors of the form **Q** = (2, 0, 0) + **q** in the pseudocubic Γ-M direction containing a diffuse scattering maximum at *T* = 546 K, in the heating cycle. The values of **q** are shown in the plot near the corresponding spectra. Solid lines show a decomposition of a spectrum into two damped harmonic oscillator contributions corresponding to the transverse acoustic and optic branches respectively. The inset shows the temperature evolution of the IC soft phonon dispersion along the ∑ direction, as reconstructed on the basis of combination of IXS result at **q** = (−0.1, 0, 0.1) and DS data. Squares correspond to *T* = 546 K, circles – to *T* = 539 K. The error bars (not shown) are estimated as 0.2 meV.

The IXS spectra for different wavevectors along the relevant ∑ direction are shown in Fig. 3. All the spectra contain maxima centred at zero energy transfer whose energy widths are larger than the energy resolution function. This implies that the scattering originates from a slow and possibly strongly damped dynamical process – a situation that was observed for the soft modes in ferroelectric perovskites^23^,^24^. The dynamic response at the most interesting wavevector **q** = (−0.2, 0, 0.2) corresponding to the DS maximum is especially slow as indicated by the narrowness of the spectrum^27^. The narrowness of the peak does not allow reliable independent analysis by fitting the spectrum. In order to estimate the energy scale of the order parameter fluctuations at this wavevector we invoke the assumption that the pattern of ionic displacements related to the IXS peak does not change strongly along the ∑ direction. The structure factor can thus be assumed approximately wavevector-independent and the magnitude of energy-integrated scattering intensity be determined mostly by the characteristic energy scale of the dynamics as *I*(**q**) ~ *T*/(*ħ*ω)^2^(**q**). It is thus possible to obtain the *ħ*ω(**q**) dependence knowing the mode energy at one of the relevant wavevectors and the integral intensity wavevector dependence. For estimating the phonon energy we have used the spectrum at **q** = (0.1, 0, 0.1), in which the two pairs of the phonon resonances are resolvable in accord with the ambient pressure results for PbZrO_3_^28^ and PbHfO_3_^29^. The damped harmonic oscillator fit of the spectrum yields the energy *E*_1_ = 3.1 ± 1 meV and damping Γ_1_ = 4.1 ± 2.5 meV for the lower-energy phonon and *E*_2_ = 7.8 ± 1.5 meV and damping Γ_2_ = 5.6 ± 3 meV for the higher-energy phonon. Using the combination of the *E*_1_ energy value and the DS data we have calculated the phonon dispersion throughout the region containing the DS maximum for *T* = 546 K and *T* = 539 K. The dependences are shown in the inset of Fig. 3. The phonon energy values at the minimum near **q** = (−0.2, 0, 0.2) are compatible with Cochran’s law (*ħω*_*IC*_)^2^ = *A*(*T* − 530 K) with constant *A* = 0.4 meV^2^/K. The value of this constant is similar to the ones for ferroelectric soft modes in perovskites^30^,^31^,^32^. The combination of DS and IXS data suggests a picture where the new non-modulated phase is characterized by soft phonon mode at and around the IC wavevector which triggers the IC phase transition and allows formation of the “missed”^7^ IC phase. This picture contrasts with the situation at ambient pressure, where there is no maximum of generalized susceptibility at a finite wavevector that allows the anharmonic interactions^7^,^9^ to trigger the formation of commensurate AFE phase instead of forming the intermediate IC phase.

Our results allow testing the modern hypothesis of AFE phase transition being driven by a ferroelectric soft mode^7^. This hypothesis gives a highly plausible description for the observed critical dynamics at ambient pressure. The ferroelectric mode, corresponding to the *q* = 0, affects *via* the flexoelectric coupling the frequency of the TA phonon branch. In this picture both the *q* = 0 TO mode and the TA branch do soften simultaneously which is in full accord with experiment at ambient pressure^7^,^28^,^33^. At high pressure the situation is different. At small wavevectors, as it is evident from Fig. 2, there is no increase of scattering intensity that would indicate the softening of the ferroelectric TO mode. The IC mode softens in the absence of the softening of ferroelectric mode, which clearly indicates that the flexoelectric interaction is not sufficient for conditioning the critical slowing down of the order parameter fluctuations along the ∑ direction and thus for the formation of the AFE phase. In the alternative explanation suggested in ref. 8 the increase of generalized susceptibility along the ∑ direction is modeled invoking the quantity of soft microscopic polarization instead of classical Landau term of macroscopic polarization, but still with the only one temperature-dependent parameter – the microscopic dielectric stiffness. This approach can reproduce a flat soft polarization branch along the ∑ direction (elasticity is neglected here) implying, again, simultaneous softening of the ferroelectric and finite-wavevector lattice modes. However, this model is neither capable of describing the experimentally observed pressure-driven change to the regime where there is only the IC lattice instability present and the ferroelectric instability is suppressed. The enhancement of finite-wavevector lattice instability in PbZrO_3_ on pressure increase is markedly different from hydrogen bonded crystals, for which the transition to ferroelectric, antiferroelectric and proton glass states is universally shifted to lower temperatures on pressure increase^34^,^35^. This makes these crystals potentially conforming to the idea of linking the zero and finite wavevector structural instabilities to the single critical (highly temperature and pressure dependent) parameter, such as macroscopic or microscopic (as in ref. 8) dielectric stiffness, while for PbZrO_3_ this is clearly not sufficient in order to describe its critical behavior.

**Figure 4:**
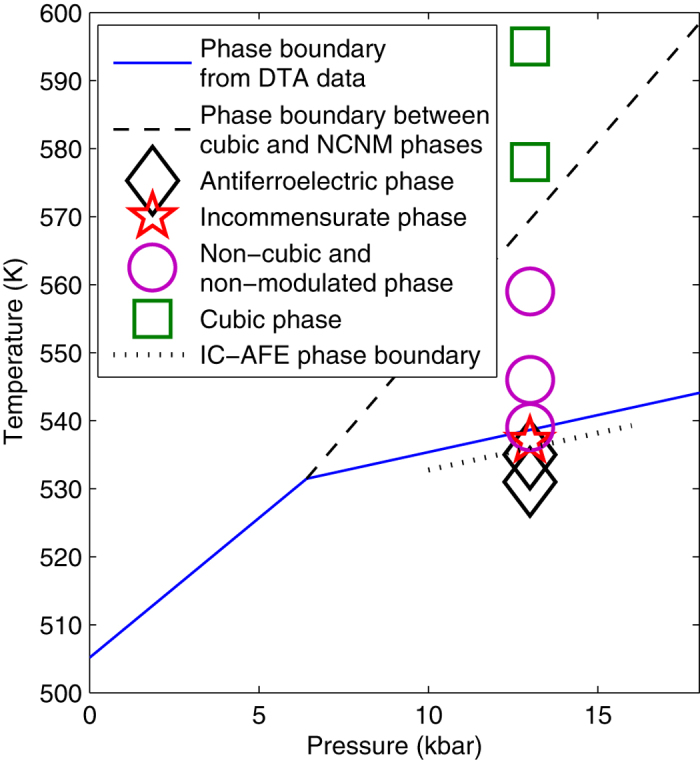
Pressure-temperature phase diagram of PbZrO_3_, obtained by correlating our x-ray scattering results at *p* = 13 kbar and the differential thermal analysis of Rapoport at variable pressure. Symbols denote the points where the x-ray measurements were performed. Solid line shows the confirmed phase boundary from DTA data, dashed and dotted lines – new phase boundaries on the basis of the present results.

The pressure-temperature phase diagram of PbZrO_3_ is much more complex than it was commonly assumed earlier. From the dielectric study of Samara^12^ it follows that for pressures at least up to 14 kbar there are no other structural transitions than the transition between cubic and antiferroelectric phases. This picture is consistent with recent first-principles study of Mani *et al*.^10^. Our data show that this picture has to be reassessed. Combined analysis of our x-ray scattering results at *p* = 13 kbar and the differential thermal analysis (DTA) of Rapoport^36^ at variable pressure allows proposing the correction to the phase diagram as shown in Fig. 4. The transition temperature from DTA data is shown by solid lines. According to the x-ray data the phase transition seen by DTA corresponds to the formation of modulations in lead sublattice. At *p* = 6.4 kbar there is a rapid change in the slope of the corresponding phase boundary. This is a triple point where the cubic phase, modulated phase and the new non-modulated phase coexist. Detecting the fact of appearance of the new non-modulated phase between cubic and modulated (IC and AFE) phases, as well as the very presence of IC phase was not possible using macroscopic methods^12^,^36^ which indicates strong demand in further studies using x-ray scattering to understand this class of complex and fascinating materials.

## Discussion

In this study we have found two new phases in antiferroelectric PbZrO_3_. One of the phases is the “missed” incommensurate phase, that was anticipated, but elusive at ambient pressure. By combining diffuse and inelastic scattering we have shown that the formation of this phase is preceded by the localized softening of a lattice mode in the vicinity of the finite wavevector, in contrast to the ambient pressure picture. However, the most surprising finding is that not only the diffuse scattering maximum appears at finite wavevector in the high-temperature phase, but the symmetry of the parent phase is lower than cubic, contrary to what is presently assumed. The pressure-temperature phase diagram is now strongly reassessed which creates the new ground for the theoretical considerations presently giving a picture inconsistent with the data under pressure^7^,^8^,^9^,^10^. Along with the new phase diagram, we have clarified the question on the possibility of antiferroelectric phase transition being driven by a single critical lattice mode and the possibility of describing the critical dynamics in PbZrO_3_ by a single critical parameter^7^,^8^. Our data clearly show that the ferroelectric softening is suppressed under pressure, while the lattice instability at finite wavevectors is enhanced. This may not be possible when there is only a single lattice instability, without strongly temperature and pressure dependent terms describing the interaction between different lattice modes. Our results show that the antiferroelectric transition is not driven by the ferroelectric soft mode and that PbZrO_3_ is not a single-instability-driven system.

## Methods

For the experiment we used PbZrO_3_ single crystals grown from high temperature solutions (flux growth method) by means of spontaneous crystallization. The Pb_3_O_4_-B_2_O_3_ mixture (soaking at 1350 K) was used as a solvent. The temperature of the melt was reduced at a rate of 3.5 K/h down to 1120 K. The remaining melt was decanted and as-grown crystals attached to the crucible walls were cooled to room temperature at a rate of 10 K/h. The as-grown crystals were colorless, but at later stages of sample preparation, the samples became slightly reddish, perhaps due to the surface modification. The phase transition temperature *T*_*c*_ was about 508–513 K, as indicated by our dielectric measurements. The single crystal sample (50 by 50 by 30 micron in size) was prepared by grinding and etching in hydrochloric acid to obtain the necessary quality of the surface for the diffuse and inelastic scattering measurements. The measurements were performed at beamline ID28^37^ of the ESRF. The single-crystal was placed in a diamond anvil cell adapted for high temperature measurements with 600 *μ*m culets which was resistively heated in a specifically designed vacuum chamber allowing the required angular flexibility for single-crystal x-ray scattering experiments. The angular opening of the chamber enables full utilization of the cell opening (70 degrees) by using air-cooled circular windows made of Kapton and metallized Mylar. The chamber itself is water cooled. Both the cell and the chamber are now available at Sample Environment Service HP, ESRF. We used rhenium as gasket material and neon as pressure transmitting medium, which allows maintaining a high level of hydrostaticity in the relevant pressure-temperature range. Pressure was measured by both Ruby and Samarium standards. The chamber was mounted on the ID28 goniometer and the analysis was carried out both by inelastic x-ray scattering (using the spectrometer) and by *in-situ* diffraction using a Pilatus 300 K area detector. This allowed assessing both the integral scattering intensity in large part of the Brillouin zone by the area detector and the energy-discriminated response by the spectrometer. We employed the silicon backscattering monochromator operating on the (999) reflection providing the beam energy of 17794 eV. The energy resolution for the incident x-rays was 2 meV, while an overall resolution of the IXS set-up was 3 meV. The resolution function was measured independently using plexiglass standard sample^38^. The beam was focused vertically by the horizontal mirror with cylindrical curvature and, afterwards, focused horizontally by the cylindrical multilayer mirror. The focusing scheme provided the spot size on the sample position about 50 by 70 micrometers.

We have studied PbZrO_3_ single crystals in a wide range of temperatures along the nearly isobaric line with pressure about 13 kbar. The pressure was 13 ± 0.3 kbar at *T* ≈ 530 K, which is on the top of the stability range of the AFE phase, and about 14 ± 1 kbar at *T* ≈ 600 K, which corresponds to the cubic phase. For the clarity of presentation we neglect this insignificant pressure variation in the text and plots.

## Additional Information

**How to cite this article**: Burkovsky, R. G. *et al*. Critical scattering and incommensurate phase transition in antiferroelectric PbZrO_3_ under pressure. *Sci. Rep.* **7**, 41512; doi: 10.1038/srep41512 (2017).

**Publisher's note:** Springer Nature remains neutral with regard to jurisdictional claims in published maps and institutional affiliations.

## Supplementary Material

Supplementary Material
