# Supplementary material for: Critical scattering and incommensurate phase transition in antiferroelectric PbZrO3 under pressure
Source: Sci Rep. 2017 Jan 30;7:41512. doi: 10.1038/srep41512 (PMC5278376; doi:10.1038/srep41512)
Supplement: Supplementary Material [file srep41512-s1.pdf]

Supplementary Materials: Critical scattering and incommensurate phase transition in antiferroelectric $PbZrO_3$ under pressure

R. G. Burkovsky,* Yu. Bronwald, and D. Andronikova
Peter the Great Saint-Petersburg Polytechnic University, 29 Politekhnicheskaya, 195251, St.-Petersburg, Russia and Ioffe Institute, 26 Politekhnicheskaya, 194021, St.-Petersburg, Russia

B. Wehinger
Department of Quantum Matter Physics, University of Geneva, 24, Quai Ernest Ansermet, 1211 Genève 4, Switzerland and Laboratory for Neutron Scattering and Imaging, Paul Scherrer Institute, 5232 Villigen PSI, Switzerland

M. Krisch, J. Jacobs, and D. Gambetti
European Synchrotron Radiation Facility, BP 220, F-38043 Grenoble Cedex, France

K. Roleder
Institute of Physics, University of Silesia, ul. Uniwersytecka 4, 40-007 Katowice, Poland

A. Majchrowski
Institute of Applied Physics, Military University of Technology, ul. Kaliskiego 2, 00-908 Warsaw, Poland

A. V. Filimonov and A. I. Rudskoy
Peter the Great Saint-Petersburg Polytechnic University, 29 Politekhnicheskaya, 195251, St.-Petersburg, Russia

S. B. Vakhrushev
Ioffe Institute, 26 Politekhnicheskaya, 194021, St.-Petersburg, Russia and Faculty of Physics, Saint-Petersburg State University, 198504 Petrodvorets, Ulyanovskaya str., 1, Saint-Petersburg, Russia

A. K. Tagantsev
Ceramics Laboratory, Swiss Federal Institute of Technology (EPFL), CH-1015 Lausanne, Switzerland and Ioffe Institute, 26 Politekhnicheskaya, 194021, St.-Petersburg, Russia

(Dated: December 15, 2016)

PACS numbers: 77.22.-d, 77.65.-j, 77.90.+k

---

* roman.burkovsky@gmail.com

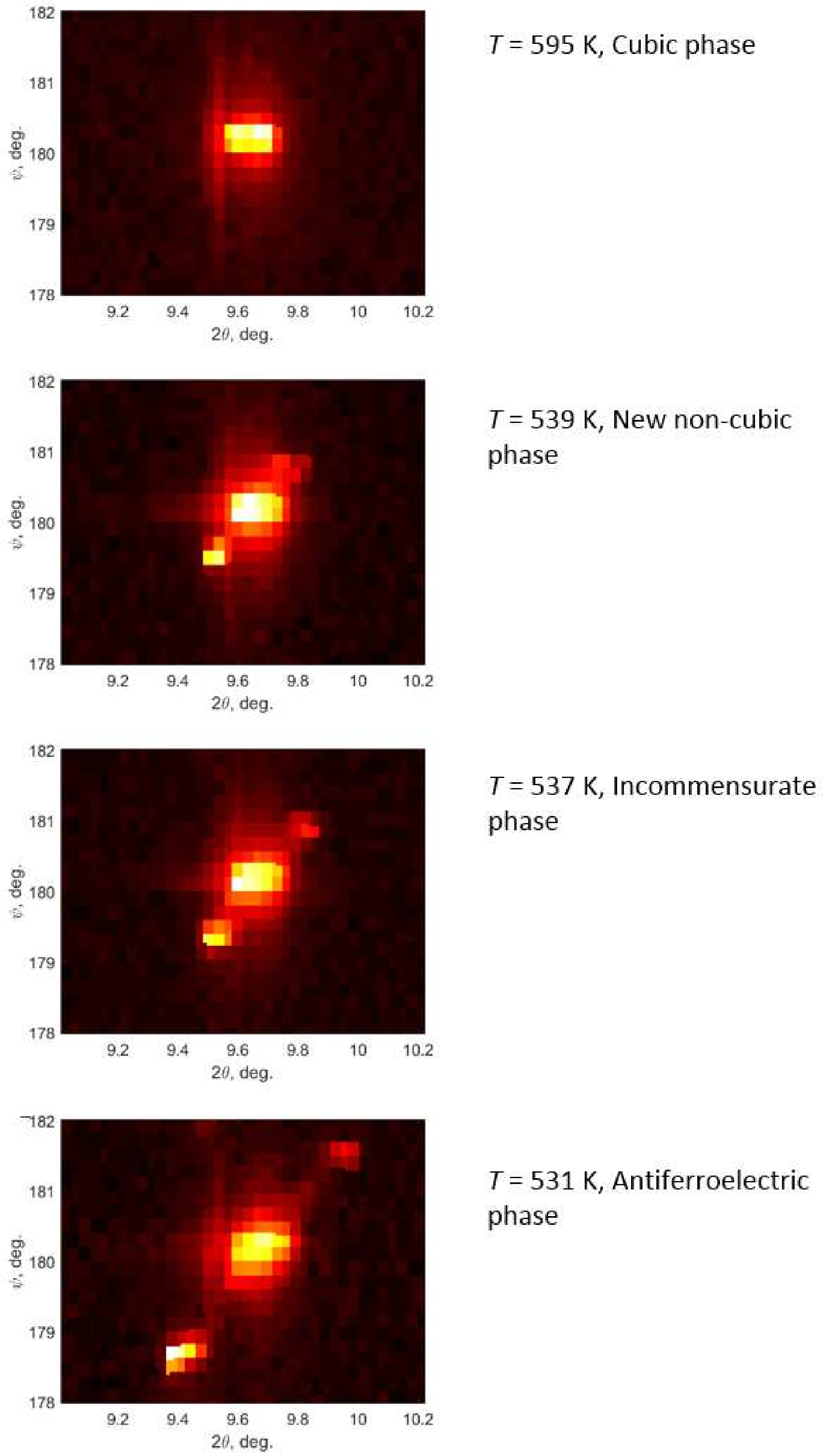

FIG. 1. Reciprocal space maps of the vicinity of the [1 0 0] pseudocubic reflection in coordinates $2\theta - \omega$ as a function of temperature. The distance, over which the pseudocubic [1 0 0] reflections corresponding to different low-symmetry domains are separated in reciprocal space, reflects the magnitude of the distortion of the unit cell shape with respect to the ideal cubic. The distortion onsets with the transition from cubic (a) to the new non-modulated phase (b) and increases consequently further on transitions to the incommensurate phase (c) and antiferroelectric phase (d).
